# Identification and Verification of the Main Differentially Expressed Proteins in Gastric Cancer via iTRAQ Combined with Liquid Chromatography-Mass Spectrometry

**DOI:** 10.1155/2019/5310684

**Published:** 2019-12-01

**Authors:** Zhihua Gao, Jiabao Wang, Yuru Bai, Jun Bao, Erqing Dai

**Affiliations:** ^1^Shenzhen Traditional Chinese Medicine Hospital, Shenzhen Guangdong 518000, China; ^2^Tianjin University of Traditional Chinese Medicine, Tianjin 301617, China; ^3^Department of Oncology, Nanjing Jiangning Hospital of Traditional Chinese Medicine, Nanjing Jiangsu 211100, China; ^4^Nanyang Traditional Chinese Medicine Hospital, Nanyang Henan 473000, China; ^5^Department of Military Medical and Health Care, Characteristic Medical Center of Chinese People's Armed Police Forces, Tianjin 300162, China

## Abstract

**Background:**

To find the potential intersections between the differentially expressed proteins and abnormally expressed genes in gastric cancer (GC) patients.

**Methods:**

Gastric cancer tissue and adjacent normal mucosa tissue were used for iTRAQ analysis. Gene ontology (GO), Kyoto Encyclopedia of Genes and Genomes (KEGG) pathway analysis, and protein-protein interaction (PPI) analysis were used to evaluate gene function. Western blotting and immunohistochemistry (IHC) were applied to verify the protein expression.

**Results:**

A total of 2770 proteins were identified, of which 147 proteins were upregulated and 159 proteins were downregulated. GO analysis revealed that the differentially expressed genes were mainly enriched for the terms “cellular process,” “binding,” and “cell.” The results of the KEGG analysis showed that the most abundantly enriched proteins were involved in the “focal adhesion” pathway. The results of the PPI analysis showed that VCAM1 was located at the center of the PPI network. Western blotting and IHC analysis demonstrated that VCAM1, FLNA, VASP, CAV1, PICK1, and COL4A2 were differentially expressed in GC and adjacent normal tissues, which was consistent with the results of the iTRAQ analysis.

**Conclusion:**

In conclusion, 6 highly differentially expressed proteins were identified as novel differentially expressed proteins in human GC. This exploratory research may provide useful information for the treatment of gastric cancer in the clinic.

## 1. Introduction

Gastric cancer (GC) is a malignant tumor originating from the gastric mucosa. It is one of the most common digestive tract tumors. China is ranked as one of countries with a high incidence of gastric cancer. There are approximately 400,000 new cases of gastric cancer diagnosed in China each year, and the death toll is approximately 350,000, which accounts for 40% of the total number of GC cases worldwide [1–3]. The early diagnosis and treatment rate of gastric cancer in China is low, and the significantly low rate of 10% is far lower than that in Japan and South Korea [4, 5]. The death rate for gastric cancer ranks third among the rates for malignant tumors. The early diagnosis and treatment rates of gastric cancer in China are relatively low, and the diagnosis of gastric cancer is made mostly in the advanced stages, resulting in a high mortality rate for gastric cancer. The early diagnosis of gastric cancer is an important step to improve the clinical curative effects of GC treatment and to save lives.

Surgery is the main treatment method for gastric cancer. Chemotherapy is the main treatment method for patients who miss the opportunity for surgery or for patients with recurrence and metastatic GC after surgery. Drug resistance (or drug insensitivity) could lead to the failure of chemotherapy, which is one of the major problems that plagues most patients during treatment [6–8]. Multidrug resistance (MDR) is the main reason for the failure of chemotherapy in gastric cancer [9]. The screening of MDR-related molecules for gastric cancer and potential markers to predict the extent of drug resistance are fundamental for the improvement of drug therapy and drug development processes. With the rapid development of genomics and proteomics, screening of the tumor target is no longer limited to subtractive hybridization and gene chip methods, and proteomics has become a new method that is used for screening tumor-related targets. One of the hot topics in proteomics research is the use of differential screening to explore the differentially expressed proteins in experimental cells (tissues) and control cells (tissues). Using this method, we explored the mediators of the upstream and downstream molecular pathways and elucidated the factors involved in the occurrence and development of disease.

The use of isobaric tags for relative and absolute quantitation with iTRAQ technology is a novel proteomics quantitative research technique used to conduct quantitative analysis in different samples simultaneously [10, 11]. iTRAQ could screen for differential proteins with good quantitative effects and high repeatability. It has become an effective method for screening differentially expressed proteins in cancer research. In this study, we examined the differentially expressed proteins in gastric cancer tissues and normal gastric mucosa using iTRAQ technology to explore the mechanism of gastric cancer.

In this study, tumor gene detection was carried out in patients to determine the potential intersections between the differentially expressed proteins and the abnormally expressed genes based on a literature search and clinical medication analysis results. This exploratory research could provide useful information for the treatment of gastric cancer in the clinic.

## 2. Methods

### 2.1. Clinical Samples

**Figure 1:**
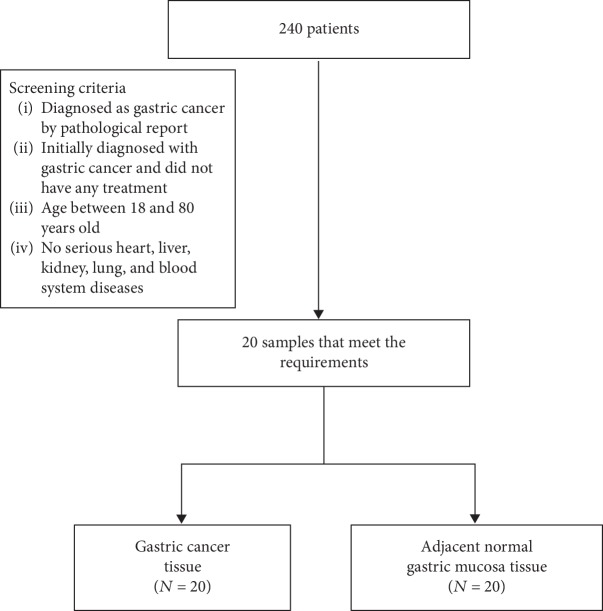
STARD flowchart of the process used to recruit the study participants.

A total of 240 GC patients were recruited from the Affiliated Hospital of the Logistics Institute of the Chinese People's Armed Police Forces between October 2014 and September 2016. All patients were diagnosed with gastric cancer by pathological examination. All patients underwent surgical resection without any prior treatment. The flow chart showing the process of the recruitment of the study participants is shown in Figure 1. After obtaining informed consent, 6 gene detections were carried out to search for the potential intersections with abnormally expressed genes and proteins in these GC patients, and the gene detection results were also used for the individually targeted treatment of the patients. The study was approved and registered with the Ethics Committee of the Affiliated Hospital of the Logistics Institute of the Chinese People's Armed Police Forces in September 2014. The Ethics Committee approved the data collection and the related screening, treatment, and follow-up of these patients. Written informed consent was obtained from all subjects. All work was undertaken according to the provisions of the Declaration of Helsinki.

### 2.2. Sample Collection and Protein Extraction

The gastric cancer tissue and adjacent normal gastric mucosa tissue were resected from GC patients. The normal gastric mucosa tissue was obtained 10-15 cm away from the tumor center and pathologically confirmed as normal gastric mucosa. The partially resected tissue was fixed with 4% formaldehyde, and the rest was stored in liquid nitrogen immediately prior to protein extraction and other follow-up analyses.

For protein extraction, the thawed tissue (150 mg) was cut into pieces with scissors. Six hundred microliters of RIPA lysis buffer (Thermo Fischer Scientific, Waltham, MA, USA) and 10 *μ*L PMSF (Thermo) were added to the tissues. The tissue was ground on ice. The suspension was mixed and processed by a homogenizer (24 × 2, 6.0 M/S, MP FastPrep-24, MP Biomedicals, Santa Ana, CA, USA) twice for 60 s. The suspension was treated by ultrasound (80 W, 10 s, 16 times) on ice and then placed in a boiling water bath for 10-15 min, followed by centrifugation at 14,000 g for 15 min. The suspension was filtered through a 0.22 *μ*m filter membrane, and the filtrate was collected. The protein quantitation of each specimen was performed by the BCA method. In the GC group or the normal control group, the samples were mixed according to the principle that the protein extracted from each specimen was added to the same amount of protein; finally, the total protein samples of the GC group and the normal control group were obtained. The total protein samples of the GC group and normal control group were collected and stored at -80°C.

### 2.3. ITRAQ Labeling

The mixed protein was reduced by alkylation and processed by enzymolysis. The sample (100 *μ*g) was labeled with iTRAQ reagents (CIEX, Framingham, MA, USA) for 2 h. The iTRAQ-labeled samples were reconstituted in 4 mL buffer A (10 mM KH_2_PO_4_ in 25% acetonitrile at pH 3.0) and loaded onto a 5 *μ*m particle size, 4.6 × 250 mm Ultremex SCX column (Phenomenex). The samples were eluted at a rate of 1 mL/min with a gradient consisting of 100% buffer A from 0 min to 25 min, 0%–10% buffer B (10 mM KH_2_PO_4_ in 25% acetonitrile/500 mM KCl at pH 3.0) from 25 min to 32 min, 10%–20% buffer B from 32 min to 42 min, 20%–45% buffer B from 42 min to 47 min, 45%–100% buffer B from 47 min to 52 min, and 100% buffer B from 52 min to 60 min. Then, the system was equilibrated with buffer A for 10 min prior to the next injection. The absorbance at 214 nm was monitored during the elution, and fractions were collected every 1 min. After lyophilization, a C18 cartridge was used for desalting.

### 2.4. nanoLC-MALDI-TOF/TOF MS/MS Assay

All samples were analyzed using the Easy nLC HPLC system (Thermo Fisher) combined with a Q Exactive mass spectrometer (Thermo Fisher). The samples were treated with a Thermo Scientific EASY Column SC200 (10 cm × 75 *μ*m, 3 *μ*m C18-A2) for 60 min with a gradient consisting of 0%-35% buffer B (84% acetonitrile/0.1% formic acid) from 0 min to 50 min, 35%-100% buffer B from 50 min to 55 min, and 100% buffer B from 55 min to 60 min. Buffer A was a 0.1% formic acid solution.

The sample was chromatographically analyzed by mass spectrometry using a Q Exactive mass spectrometer. The analysis time was 60 min, and the detection mode was positive ion mode. The parent ion scanning ranged from 300 *m*/*z* to 1800 *m*/*z*. The primary mass spectrometer resolution was 70,000 at 200 *m*/*z*. The AGC (automatic gain control) target was 1e6, and the maximum IT was 50 ms. The dynamic exclusion was 60.0 s. The mass-to-charge ratio of the polypeptide and polypeptide fragments was determined using the following parameters: 20 fragments were acquired after each full scan, the MS2 activation type was HCD, the isolation window was 2 *m*/*z*, the secondary mass spectrometer resolution was 17,500 at 200 *m*/*z*, the normalized collision energy was 30 eV, and the underfill was 0.1%. Mascot 2.2 and Proteome Discoverer 1.4 software were used for the data analysis.

### 2.5. Gene Ontology (GO), Protein-Protein Interaction (PPI), and Kyoto Encyclopedia of Genes and Genomes (KEGG) Pathway Analyses

The differentially expressed genes were annotated using the Database for Annotation, Visualization, and Integrated Discovery (DAVID; http://david.ncifcrf.gov) (version 6.7), and the enriched biological metabolic pathways were determined using the Kyoto Encyclopedia of Genes and Genomes (KEGG) (http://www.genome.jp/kegg/). A *P* value < 0.05 was considered to indicate a significant correlation. The PPI network was assessed using the Search Tool for the Retrieval of Interacting Genes database (STRING, https://string-db.org/) and visualized using Cytoscape software according to the previous reference [12–14].

### 2.6. Western Blotting

Total proteins were extracted using RIPA lysis buffer (Pierce, Invitrogen, Gaithersburg, MD, USA). The concentration of the extracted protein was determined by a BCA assay. The total protein was separated by 12% sodium dodecyl sulfate-polyacrylamide gel electrophoresis (SDS-PAGE), followed by transfer to a PVDF membrane (EMD Millipore, Billerica, MA, USA), which was blocked with 5% skim milk for 1 h. The primary antibodies, anti-VCAM1 (1 : 1000 dilution, Cell Signaling Technology, MA, USA), anti-VASP (1 : 1000 dilution, Cell Signaling Technology), anti-PICK1 (1 : 1000 dilution, Cell Signaling Technology), anti-FLNA (1 : 1000 dilution, Cell Signaling Technology), anti-COL4A2 (1 : 1000 dilution, Cell Signaling Technology), anti-CAV1 (1 : 1000 dilution, Cell Signaling Technology), and anti-GAPDH (1 : 1000 dilution, Cell Signaling Technology), were added and incubated with the membranes at 4°C overnight. Then, the membranes were washed with PBS buffer and incubated with anti-rabbit IgG antibody (1 : 10,000 dilution, Cell Signaling Technology) at 37°C for 45 min. An imaging system (Odyssey, LI-COR Biosciences, Lincoln, NE, USA) was used for the semiquantitative analysis. GAPDH was used as an internal control.

### 2.7. Immunohistochemistry (IHC) Assay

GC and control samples were fixed with 4% formaldehyde solution and embedded in paraffin. Then, the sections (5 *μ*m thickness) were incubated with 3% H_2_O_2_ for 10 min at room temperature to eliminate endogenous peroxidase activity. The sections were blocked with 10% goat serum at room temperature for 10 min. Then, the sections were incubated with primary antibodies, including anti-CAV1 (1 : 1000 dilution, Cell Signaling Technology), anti-VASP (1 : 1000 dilution, Cell Signaling Technology), and anti-VCAM1 (1 : 1000 dilution, Cell Signaling Technology, MA, USA), at 37°C for 2 h. After the application of the secondary antibody (anti-rabbit IgG antibody, 1 : 10,000 dilution, Cell Signaling Technology), the sections were incubated at 37°C for 30 min. Subsequently, the DAB Plus Substrate Chromogen mixture was added, and the sections were incubated for 10 min.

The Human Protein Atlas (http://www.proteinatlas.org/) was used to validate the expression of the six genes in GC tissue.

### 2.8. Statistical Analysis

SPSS 22.0 statistical software was used for the statistical analysis. The values were expressed as the mean ± standard deviation (SD) and compared using Student's *t* test or the Wilcoxon/Mann-Whitney rank sum test. *P* < 0.05 was considered to indicate a statistically significant difference.

## 3. Results

### 3.1. iTRAQ and GO Analysis

**Figure 2:**
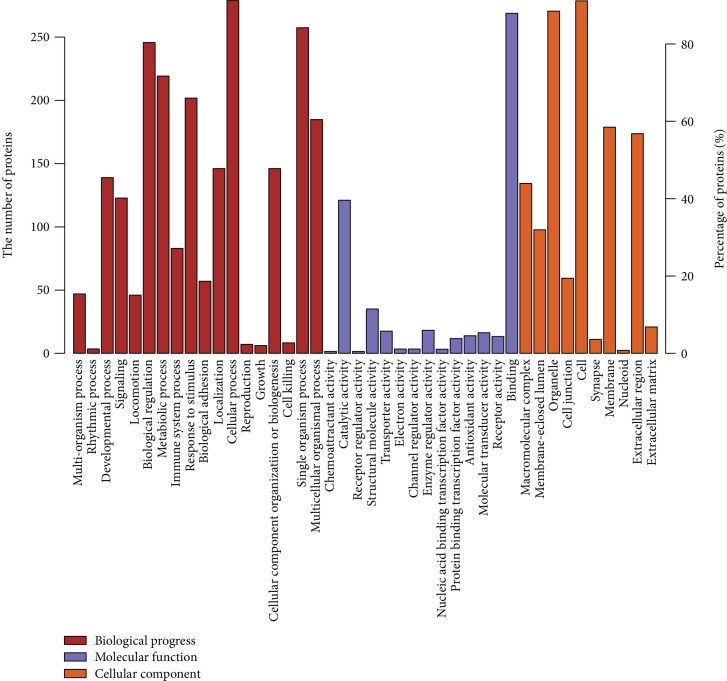
GO analysis of the differentially expressed genes. The differentially expressed proteins were enriched in molecular function (MF), biological process (BP), and cellular component (CC) terms.

Compared to adjacent normal tissues, 2770 proteins were differentially expressed in tumor tissues, of which 147 were upregulated by more than 1.2-fold (*P* < 0.05) and 159 were downregulated by more than 0.8-fold (*P* < 0.05). The top 50 proteins upregulated by more than 1.2-fold and downregulated by more than 0.8-fold are shown in Tables 1 and 2, respectively. Subsequently, GO analysis was applied to analyze the differentially expressed genes. The differentially expressed genes were enriched in various molecular functions (MF), biological processes (BP), and cellular component terms (CC) (Figure 2). “Cellular process,” “binding,” and “cell” were the most enriched terms in BP, MF, and CC, respectively (Figure 2).

### 3.2. KEGG Pathway and Protein-Protein Interaction (PPI) Analysis

**Figure 3:**
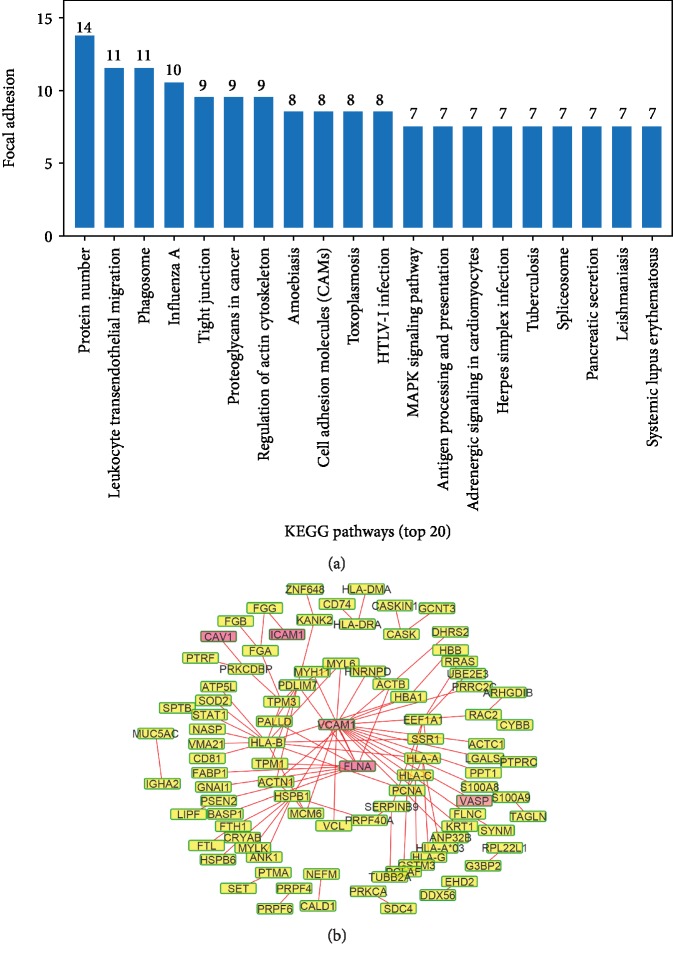
KEGG pathway and PPI analysis of the differentially expressed proteins. (a) The top 20 KEGG pathways with differentially expressed protein enrichment. (b) VCAM1 was located at the center of the PPI network.

The KEGG analysis revealed differential protein enrichment in 41 KEGG metabolic pathways (Table 3). The top 20 metabolic pathways are shown in Figure 3(a), and the most abundantly enriched protein was involved in the “focal adhesion” pathway. The differentially expressed proteins involved in “focal adhesion” pathways included COL6A3, MYLK, VASP, FLNC, FLNA, ACTN2, PARVA, ACTN1, ITGA5, CAV1, VCL, PICK1, COL4A2, and ITGA1. The detailed information about these 14 proteins is listed in Table 4. In addition, the results of the PPI analysis showed that VCAM1 was located at the center of the PPI network (Figure 3(b)).

### 3.3. Verification of the Differentially Expressed Proteins Involved in the “Focal Adhesion” Pathway and Located at the Center of the PPI Network

**Figure 4:**
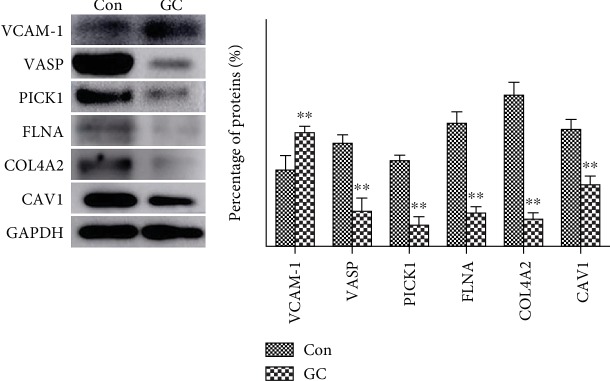
Western blotting was performed to measure the levels of VCAM1, FLNA, VASP, CAV1, PICK1, and COL4A2 in GC and adjacent normal tissues. ^∗∗^*P* < 0.01.

**Figure 5:**
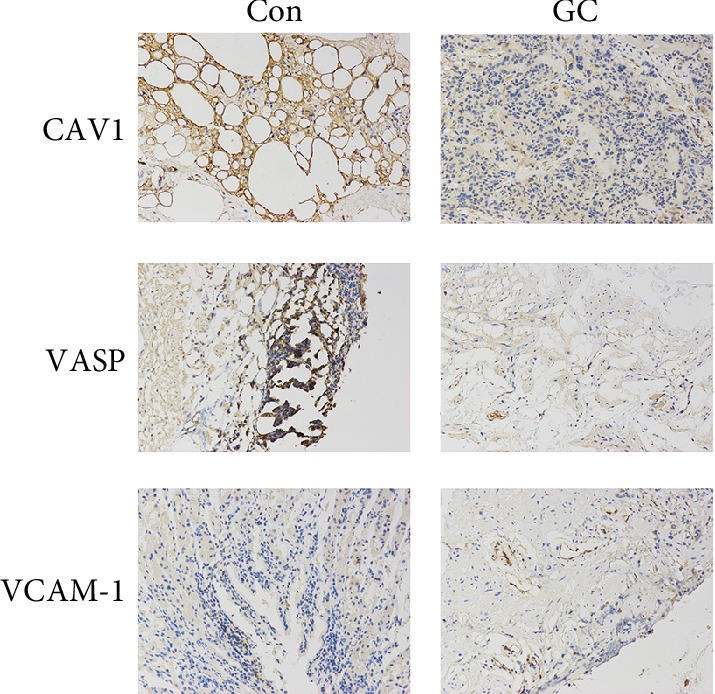
The expression levels of CAV1, VASP, and VCAM1 in GC and adjacent normal tissues were verified by IHC assays. ^∗∗∗^*P* < 0.001.

**Figure 6:**
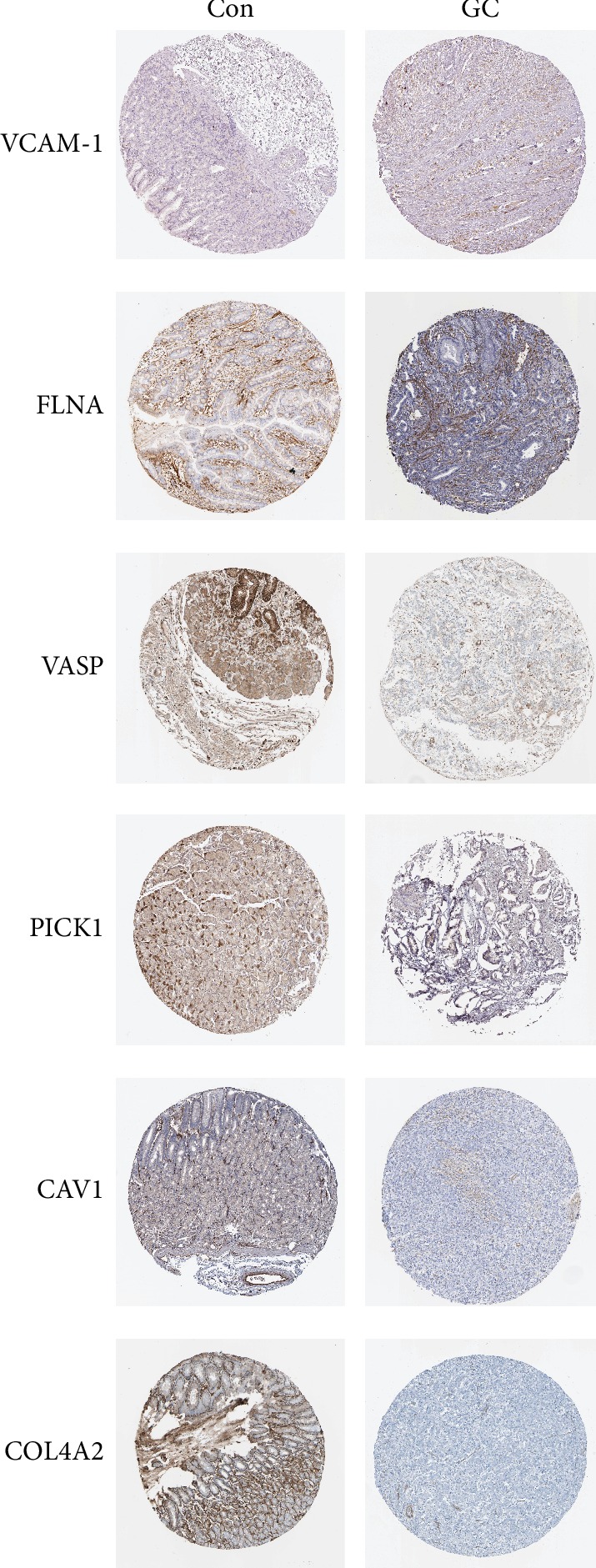
The protein levels of VCAM1, FLNA, VASP, CAV1, PICK1, and COL4A2 in GC and adjacent normal tissue. Images were obtained from the Human Protein Atlas (http://www.proteinatlas.org/).

**Figure 7:**
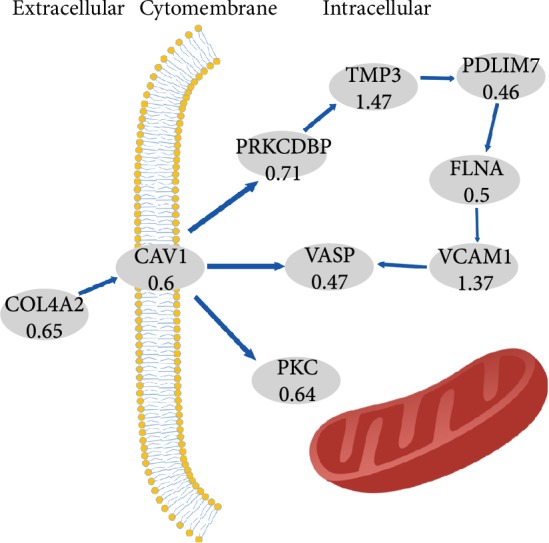
A simple activity flowchart of the main differentially expressed proteins.

Western blot assays were performed to measure the expression levels of VCAM1 and 5 other proteins (FLNA, VASP, CAV1, PICK1, and COL4A2) enriched in the “focal adhesion” pathway in GC and adjacent normal tissues. As shown in Figure 4, the results of Western blotting were consistent with the trends revealed by the iTRAQ assay. Furthermore, the expression levels of VASP (highest enrichment in the “focal adhesion” pathway), VCAM1 (located at the center of the PPI network) and CAV1 (related to the metastasis, proliferation, and aggregation of GC cells) were detected by IHC. The results showed that compared with their expression levels in adjacent normal tissues, CAV1 and VASP were downregulated in GC tissues (*P* < 0.001, Figure 5), while VCAM1 was upregulated in GC tissues (*P* < 0.001, Figure 5). For validation of the identified differentially expressed proteins, the Human Protein Atlas database was searched to analyze the expression of VCAM1, FLNA, VASP, CAV1, PICK1, and COL4A2 in GC and adjacent normal tissues. As shown in Figure 6, the trends were consistent with the results of Western blotting and iTRAQ analysis. We speculated that the downregulation of COL4A2 outside the cell may downregulate the expression of CAV1 in the cell membrane through cell signaling, thereby affecting the intracellular expression of FLNA, VASP, and PICK1. We generated a simple activity flowchart of these proteins in Figure 7.

## 4. Discussion

In this study, most of the patients were already diagnosed with advanced gastric cancer. Their pathological differentiation was poor. Differentiated tumor cells have significant differences compared to normal gastric mucosa cells. The proliferation and differentiation abilities of these immature tumor cells were much higher than those of early gastric cancer cells. Thus, the overall condition and prognosis of the patients were poor. Due to recent progress in drug treatment, the therapeutic effect of chemotherapy, especially targeted drugs, in the treatment of gastric cancer has been improved. However, there are still many clinical problems that need to be solved. We collected the resected GC samples, tested the differentially expressed proteins and genes in response to individual treatments, and attempted to explore the occurrence and development of tumors from the perspective of proteomics and gene changes and to determine the interactions between proteins and genes. We found only an interaction between TOPO IIa and filamin A (FLNA), and no other intersection has yet been found.

We speculate that the main reason for this is that we did not analyze these resected samples according to their Lauren classification. The GC samples from recruited patients were combined according to intestinal type, diffuse type, and mixed type for the iTRAQ analysis. Tan et al. [15] analyzed the gene expression profiles of 37 gastric cancer cell lines. They finally found 171 gene chips and divided them into the gastric intestinal (G-INT) and gastric diffuse subtypes (G-DIF). Further *in vitro* drug sensitivity tests demonstrated that cells of the G-INT type are sensitive to 5-FU and oxaliplatin. In addition, cells of the G-DIF type are sensitive to cisplatin. However, based on the pathological types of gastric cancer and the use of genotyping to guide evidence-based medicine, treatment options are very limited. However, this is the only method available for the individualized treatment of gastric cancer.

To date, there are several mechanisms of multidrug resistance (MDR) in tumors that have been identified. (1) Intracellular drugs are discharged to the outside of the cell membrane by the ABC (ATP-binding cassette) transporter protein family, and the accumulation of intracellular drugs is reduced. (2) The cytotoxicity of chemotherapy drugs is reduced by multiple detoxification molecules. (3) The concentration of drugs is reduced by exocytosis in cells. (4) The abnormal distribution or the change in the number of molecular targets causes drugs to lose their function. (5) The antiapoptotic ability of tumors is enhanced by molecular apoptosis.

FLNA, also called filamin A, plays important roles in the formation and function of the cytoskeleton. Studies have demonstrated that the FLNA protein may interact with multiple proteins and take part in the development of tumors [16, 17]. Our iTRAQ results showed that the expression of FLNA in GC samples was decreased by 0.502-fold compared with that in normal adjacent samples. Lv et al. [18] also showed that the expression of FLNA in GC tissues is lower than that in adjacent tissues, which is consistent with the results of our study. Their research also indicated that the survival of the FLNA low-expression group was significantly lower than that of the FLNA high-expression group. Zhai et al. [19] observed that the proliferation, invasion, and metastasis ability of hepatocellular carcinoma, colorectal cancer cells, and nasopharyngeal carcinoma cells were significantly reduced when FLNA was highly expressed.

Zhao et al. [20] indicated that xenografted mice with FLNA knockdown showed an enhanced response to docetaxel compared with control xenografted mice with increased apoptosis. Topoisomerase II (TOPO II A) is located in the nucleus of human cells and is a critical enzyme involved in biological behavior, such as DNA replication, transcription, translation, repair, and recombination, chromosome segregation, and nucleic acid conformation [21]. Reports have indicated that the expression of TOPO II A is related to tumor growth and stage, the invasion of tumor cells into the surrounding tissue, and the metastasis of the tumor. Uesaka et al. [22] demonstrated that patients with high expression of TOPO II mRNA are more sensitive to etoposide. Lu et al. [23] showed that the gene is a crucial mediator of apoptosis triggered by doxorubicin. FILIP1L levels were increased markedly through transcriptional mechanisms following treatment with doxorubicin and other TOP2 inhibitors, including etoposide and mitoxantrone, but not the TOP2 catalytic inhibitors merbarone or dexrazoxane. These results indicate that the FILIP1L expression status in tumors may influence the response to anti-TOP2 chemotherapeutics. These studies imply that FLNA might participate in drug resistance to chemotherapy via its enhanced antiapoptosis ability.

Vasodilator-stimulated phosphoprotein (VASP) plays an important role in the three-dimensional structure of actin protein and participates in the process of cell migration. VASP is involved in tumor invasion and/or metastasis progression [24]. COL4A2 is involved in tight junctions between a variety of human cells and plays a role in the adhesion of cancer cells [25]. Our results showed that the expression of VASP and COL4A2 in GC tissue was decreased compared with that in normal adjacent tissues. We speculate that the decreased expression of VASP reduced the adhesion and aggregation of tumor cells, which may lead to the invasion and metastasis of tumor cells into their surroundings. In addition, the expression of CAV1 (caveolin 1) and VCAM-1 (vascular cell adhesion molecule-1) in GC tissue was also consistent with that described in previous reports [26, 27]. However, the expression of PICK1 (protein kinase C alpha) was upregulated in our research, which is entirely different from the results of Sun et al.'s research [28]. This might be because the expression of PICK1 is related to the stage of GC, and the mixed samples used in our research were different from the samples they used, which led to different results.

In conclusion, we investigated the differential protein expression in gastric cancer tissues and normal gastric mucosa using iTRAQ technology to explore the mechanism involved in gastric cancer. Six highly differentially expressed proteins were screened to identify the potential intersections between the differentially expressed proteins and abnormally expressed genes. This exploratory research may provide useful information for the clinical treatment of GC.

## Figures and Tables

**Table 1:** The top 50 proteins with upregulation multiples greater than 1.2-fold.

N	Accession	Description	Up fold	*P* value
1	A0A087X079	Ig gamma-1 chain C region OS=Homo sapiens GN=IGHG1 PE=1 SV=1 (A0A087X079_HUMAN)	4.683184	1.29*E*-25
2	O14604	Thymosin beta-4, Y-chromosomal OS=Homo sapiens GN=TMSB4Y PE=1 SV=3 (TYB4Y_HUMAN)	3.016063	8.25*E*-14
3	P31327	Carbamoyl-phosphate synthase (ammonia), mitochondrial OS=Homo sapiens GN=CPS1 PE=1 SV=2 (CPSM_HUMAN)	2.721231	1.33*E*-11
4	A1A5C5	RRBP1 protein OS=Homo sapiens GN=RRBP1 PE=2 SV=1 (A1A5C5_HUMAN)	2.547532	2.67*E*-10
5	P25815	Protein S100-P OS=Homo sapiens GN=S100P PE=1 SV=2 (S100P_HUMAN)	2.528605	3.71*E*-10
6	P0DMU9	Cancer/testis antigen family 45 member A10 OS=Homo sapiens GN=CT45A10 PE=2 SV=1 (CT45A_HUMAN)	2.486112	7.72*E*-10
	Q86XP6	Gastrokine-2 OS=Homo sapiens GN=GKN2 PE=1 SV=2 (GKN2_HUMAN)	2.283884	2.51*E*-08
8	B3KY79	cDNA FLJ46620 fis, clone TLUNG2000654, highly similar to keratin, type II cytoskeletal 7 OS=Homo sapiens PE=2 SV=1 (B3KY79_HUMAN)	2.215252	8.09*E*-08
9	A8K2T5	cDNA FLJ77047, highly similar to Homo sapiens zinc finger protein 217 (ZNF217), mRNA (fragment) OS=Homo sapiens PE=2 SV=1 (A8K2T5_HUMAN)	2.209209	8.97*E*-08
10	B4E2T6	cDNA FLJ58231, highly similar to NMDA receptor-regulated protein 1 OS=Homo sapiens PE=2 SV=1 (B4E2T6_HUMAN)	2.045548	1.42*E*-06
11	D6CHE9	Proteinase 3 OS=Homo sapiens GN=PRTN3 PE=2 SV=1 (D6CHE9_HUMAN)	2.038724	1.59*E*-06
12	H0YJL6	Ena/VASP-like protein (fragment) OS=Homo sapiens GN=EVL PE=1 SV=1 (H0YJL6_HUMAN)	2.036708	1.64*E*-06
13	Q5T619	Zinc finger protein 648 OS=Homo sapiens GN=ZNF648 PE=2 SV=1 (ZN648_HUMAN)	2.035727	1.67*E*-06
14	Q658S4	Putative uncharacterized protein DKFZp666N164 (fragment) OS=Homo sapiens GN=DKFZp666N164 PE=2 SV=1 (Q658S4_HUMAN)	1.990709	3.53*E*-06
15	B2RBL3	Thymidine phosphorylase OS=Homo sapiens PE=2 SV=1 (B2RBL3_HUMAN)	1.988453	3.66*E*-06
16	P98088	Mucin-5AC OS=Homo sapiens GN=MUC5AC PE=1 SV=4 (MUC5A_HUMAN)	1.981462	4.11*E*-06
17	Q9HD89	Resistin OS=Homo sapiens GN=RETN PE=1 SV=1 (RETN_HUMAN)	1.949174	6.99*E*-06
18	B4DLS7	cDNA FLJ53454, highly similar to interferon-induced protein with tetratricopeptide repeats 3 OS=Homo sapiens PE=2 SV=1 (B4DLS7_HUMAN)	1.911231	1.30*E*-05
19	P06702	Protein S100-A9 OS=Homo sapiens GN=S100A9 PE=1 SV=1 (S10A9_HUMAN)	1.882936	2.06*E*-05
20	C9JKF7	Lymphocyte-specific protein 1 (fragment) OS=Homo sapiens GN=LSP1 PE=1 SV=1 (C9JKF7_HUMAN)	1.878033	2.23*E*-05
21	P52566	Rho GDP-dissociation inhibitor 2 OS=Homo sapiens GN=ARHGDIB PE=1 SV=3 (GDIR2_HUMAN)	1.843884	3.86*E*-05
22	P16402	Histone H1.3 OS=Homo sapiens GN=HIST1H1D PE=1 SV=2 (H13_HUMAN)	1.834376	4.49*E*-05
23	Q7Z351	Putative uncharacterized protein DKFZp686N02209 OS=Homo sapiens GN=DKFZp686N02209 PE=2 SV=1 (Q7Z351_HUMAN)	1.82681	5.07*E*-05
24	D3DP16	Fibrinogen gamma chain, isoform CRA_a OS=Homo sapiens GN=FGG PE=4 SV=1 (D3DP16_HUMAN)	1.818705	5.77*E*-05
25	P40261	Nicotinamide N-methyltransferase OS=Homo sapiens GN=NNMT PE=1 SV=1 (NNMT_HUMAN)	1.792604	8.72*E*-05
26	B7Z747	cDNA FLJ51120, highly similar to matrix metalloproteinase-9 (EC 3.4.24.35) OS=Homo sapiens PE=2 SV=1 (B7Z747_HUMAN)	1.756301	0.000154
27	I1VZV6	Hemoglobin alpha 1 OS=Homo sapiens GN=HBA1 PE=3 SV=1 (I1VZV6_HUMAN)	1.755144	0.000157
28	P02792	Ferritin light chain OS=Homo sapiens GN=FTL PE=1 SV=2 (FRIL_HUMAN)	1.75021	0.000169
29	F5H5I5	ATP-binding cassette sub-family B member 9 (fragment) OS=Homo sapiens GN=ABCB9 PE=4 SV=1 (F5H5I5_HUMAN)	1.746098	0.000181
30	Q6P4A8	Phospholipase B-like 1 OS=Homo sapiens GN=PLBD1 PE=1 SV=2 (PLBL1_HUMAN)	1.732104	0.000224
31	H0YJG9	Dehydrogenase/reductase SDR family member 2, mitochondrial (fragment) OS=Homo sapiens GN=DHRS2 PE=1 SV=1 (H0YJG9_HUMAN)	1.727996	0.000239
32	C9JZJ5	Melanoma-associated antigen 4 (fragment) OS=Homo sapiens GN=MAGEA4 PE=1 SV=7 (C9JZJ5_HUMAN)	1.717063	0.000283
33	P20591	Interferon-induced GTP-binding protein Mx1 OS=Homo sapiens GN=MX1 PE=1 SV=4 (MX1_HUMAN)	1.705117	0.00034
34	Q400J6	Arylamine N-acetyltransferase (fragment) OS=Homo sapiens GN=NAT1 PE=2 SV=1 (Q400J6_HUMAN)	1.6983	0.000377
35	P80723	Brain acid soluble protein 1 OS=Homo sapiens GN=BASP1 PE=1 SV=2 (BASP1_HUMAN)	1.696699	0.000386
36	P05109	Protein S100-A8 OS=Homo sapiens GN=S100A8 PE=1 SV=1 (S10A8_HUMAN)	1.684961	0.000462
37	B2MUD5	Neutrophil elastase (fragment) OS=Homo sapiens GN=ELA2 PE=4 SV=1 (B2MUD5_HUMAN)	1.681383	0.000487
38	P23381	Tryptophan-tRNA ligase, cytoplasmic OS=Homo sapiens GN=WARS PE=1 SV=2 (SYWC_HUMAN)	1.670288	0.000576
39	Q86YQ1	Hemoglobin alpha-2 (fragment) OS=Homo sapiens GN=HBA2 PE=3 SV=1 (Q86YQ1_HUMAN)	1.669802	0.000581
40	A0A0G2JMH6	HLA class II histocompatibility antigen, DR alpha chain OS=Homo sapiens GN=HLA-DRA PE=1 SV=1 (A0A0G2JMH6_HUMAN)	1.663235	0.000641
41	B4DVG3	cDNA FLJ53104, moderately similar to Homo sapiens N-acetylneuraminate pyruvate lyase (dihydrodipicolinate synthase) (NPL), mRNA OS=Homo sapiens PE=2 SV=1 (B4DVG3_HUMAN)	1.662357	0.000649
42	A0A140VJJ6	Testicular tissue protein Li 70 OS=Homo sapiens PE=2 SV=1 (A0A140VJJ6_HUMAN)	1.653489	0.000742
43	B4E0J9	cDNA FLJ57348, highly similar to Homo sapiens hexokinase domain containing 1 (HKDC1), mRNA OS=Homo sapiens PE=2 SV=1 (B4E0J9_HUMAN)	1.653261	0.000744
44	U6FVB0	Tyrosine-protein kinase receptor OS=Homo sapiens GN=CD74-Ntrk1 fusion gene PE=2 SV=1 (U6FVB0_HUMAN)	1.616527	0.001283
45	A2MYE1	A30 (fragment) OS=Homo sapiens PE=4 SV=1 (A2MYE1_HUMAN)	1.599301	0.001651
46	B4DVC2	cDNA FLJ51332, highly similar to HLA class II histocompatibility antigen, DMbeta chain OS=Homo sapiens PE=2 SV=1 (B4DVC2_HUMAN)	1.592926	0.001811
47	Q8IZI0	Hemoglobin beta chain variant Hb-I_Toulouse (fragment) OS=Homo sapiens GN=HBB PE=3 SV=1 (Q8IZI0_HUMAN)	1.592452	0.001823
48	Q6P1N7	TAPBP protein OS=Homo sapiens GN=TAPBP PE=1 SV=1 (Q6P1N7_HUMAN)	1.584238	0.002054
49	B4DNT5	cDNA FLJ60316, highly similar to Apolipoprotein-L1 OS=Homo sapiens PE=2 SV=1 (B4DNT5_HUMAN)	1.577475	0.002264
50	O15451	Proline and glutamic acid-rich nuclear protein isoform (fragment) OS=Homo sapiens PE=2 SV=2 (O15451_HUMAN)	1.576906	0.002282

**Table 2:** The top 50 proteins with downregulation folds less than 0.8-fold.

n	Accession	Description	Down fold	*P* value
1	O60844	Zymogen granule membrane protein 16 OS=Homo sapiens GN=ZG16 PE=1 SV=2 (ZG16_HUMAN)	0.198959	3.21*E*-19
2	E1CKY7	Protein phosphatase 1 regulatory subunit 12B OS=Homo sapiens GN=sm-M20 PE=1 SV=1 (E1CKY7_HUMAN)	0.254236	2.81*E*-14
3	Q9BYX7	Putative beta-actin-like protein 3 OS=Homo sapiens GN=POTEKP PE=5 SV=1 (ACTBM_HUMAN)	0.260689	8.13*E*-14
4	P51911	Calponin-1 OS=Homo sapiens GN=CNN1 PE=1 SV=2 (CNN1_HUMAN)	0.262319	1.06*E*-13
5	B7Z9B7	cDNA FLJ54732, moderately similar to sorbin and SH3 domain-containing protein 1 OS=Homo sapiens PE=2 SV=1 (B7Z9B7_HUMAN)	0.315502	1.42*E*-10
6	B4E1Q7	cDNA FLJ57294, highly similar to lipoamide acyltransferase component of branched-chain alpha-keto acid dehydrogenase complex, mitochondrial (EC 2.3.1.168) OS=Homo sapiens PE=2 SV=1 (B4E1Q7_HUMAN)	0.339832	1.93*E*-09
7	Q9BTA4	Epididymis secretory protein Li 286 (fragment) OS=Homo sapiens GN=HEL-S-286 PE=2 SV=1 (Q9BTA4_HUMAN)	0.342304	2.47*E*-09
8	A5Z217	Mutant desmin OS=Homo sapiens PE=2 SV=1 (A5Z217_HUMAN)	0.382163	8.56*E*-08
9	A5A3E0	POTE ankyrin domain family member F OS=Homo sapiens GN=POTEF PE=1 SV=2 (POTEF_HUMAN)	0.387963	1.35*E*-07
10	Q96JG9	Zinc finger protein 469 OS=Homo sapiens GN=ZNF469 PE=2 SV=3 (ZN469_HUMAN)	0.395397	2.38*E*-07
11	P08217	Chymotrypsin-like elastase family member 2A OS=Homo sapiens GN=CELA2A PE=1 SV=1 (CEL2A_HUMAN)	0.398866	3.08*E*-07
12	P07098	Gastric triacylglycerol lipase OS=Homo sapiens GN=LIPF PE=1 SV=1 (LIPG_HUMAN)	0.405473	4.96*E*-07
13	B7Z6U8	cDNA FLJ53665, highly similar to four and a half LIM domains protein 1 OS=Homo sapiens PE=2 SV=1 (B7Z6U8_HUMAN)	0.408392	6.09*E*-07
14	P12277	Creatine kinase B-type OS=Homo sapiens GN=CKB PE=1 SV=1 (KCRB_HUMAN)	0.409014	6.36*E*-07
15	P0CG38	POTE ankyrin domain family member I OS=Homo sapiens GN=POTEI PE=3 SV=1 (POTEI_HUMAN)	0.409772	6.71*E*-07
16	B7Z7M8	cDNA FLJ60950, highly similar to hydroxymethylglutaryl-CoA synthase, mitochondrial (EC 2.3.3.10) OS=Homo sapiens PE=2 SV=1 (B7Z7M8_HUMAN)	0.419918	1.33*E*-06
17	P35749	Myosin-11 OS=Homo sapiens GN=MYH11 PE=1 SV=3 (MYH11_HUMAN)	0.420775	1.41*E*-06
18	O15061	Synemin OS=Homo sapiens GN=SYNM PE=1 SV=2 (SYNEM_HUMAN)	0.430091	2.57*E*-06
19	P68032	Actin, alpha cardiac muscle 1 OS=Homo sapiens GN=ACTC1 PE=1 SV=1 (ACTC_HUMAN)	0.437479	4.06*E*-06
20	P26678	Cardiac phospholamban OS=Homo sapiens GN=PLN PE=1 SV=1 (PPLA_HUMAN)	0.440292	4.81*E*-06
21	Q15124	Phosphoglucomutase-like protein 5 OS=Homo sapiens GN=PGM5 PE=1 SV=2 (PGM5_HUMAN)	0.442845	5.60*E*-06
22	B4DTX5	cDNA FLJ60072, highly similar to Homo sapiens sorbin and SH3 domain containing 1 (SORBS1), transcript variant 6, mRNA OS=Homo sapiens PE=2 SV=1 (B4DTX5_HUMAN)	0.444007	6.00*E*-06
23	B3KW93	Sodium/potassium-transporting ATPase subunit alpha OS=Homo sapiens PE=2 SV=1 (B3KW93_HUMAN)	0.446979	7.14*E*-06
24	F8VPF3	Myosin light polypeptide 6 (fragment) OS=Homo sapiens GN=MYL6 PE=1 SV=1 (F8VPF3_HUMAN)	0.448822	7.94*E*-06
25	Q9NR12	PDZ and LIM domain protein 7 OS=Homo sapiens GN=PDLIM7 PE=1 SV=1 (PDLI7_HUMAN)	0.455034	1.13*E*-05
26	Q01995	Transgelin OS=Homo sapiens GN=TAGLN PE=1 SV=4 (TAGL_HUMAN)	0.456778	1.24*E*-05
27	A0A024R5W6	Tropomyosin 1 (alpha), isoform CRA_a OS=Homo sapiens GN=TPM1 PE=3 SV=1 (A0A024R5W6_HUMAN)	0.458474	1.37*E*-05
28	Q63ZY3	KN motif and ankyrin repeat domain-containing protein 2 OS=Homo sapiens GN=KANK2 PE=1 SV=1 (KANK2_HUMAN)	0.461142	1.58*E*-05
29	E9PIE4	Mitochondrial carrier homolog 2 (fragment) OS=Homo sapiens GN=MTCH2 PE=1 SV=7 (E9PIE4_HUMAN)	0.462079	1.66*E*-05
30	Q16853	Membrane primary amine oxidase OS=Homo sapiens GN=AOC3 PE=1 SV=3 (AOC3_HUMAN)	0.465486	2.00*E*-05
31	Q15746	Myosin light chain kinase, smooth muscle OS=Homo sapiens GN=MYLK PE=1 SV=4 (MYLK_HUMAN)	0.466825	2.14*E*-05
32	B3KUD6	cDNA FLJ39634 fis, clone SMINT2002689, highly similar to SMOOTHELIN OS=Homo sapiens PE=2 SV=1 (B3KUD6_HUMAN)	0.467199	2.19*E*-05
33	A0A024R5N4	WD repeat domain 71, isoform CRA_a OS=Homo sapiens GN=WDR71 PE=4 SV=1 (A0A024R5N4_HUMAN)	0.467661	2.24*E*-05
34	P68133	Actin, alpha skeletal muscle OS=Homo sapiens GN=ACTA1 PE=1 SV=1 (ACTS_HUMAN)	0.467865	2.26*E*-05
35	K7EM16	Vasodilator-stimulated phosphoprotein (fragment) OS=Homo sapiens GN=VASP PE=1 SV=1 (K7EM16_HUMAN)	0.471806	2.78*E*-05
36	B4DWU6	cDNA FLJ51361, highly similar to keratin, type II cytoskeletal 6A OS=Homo sapiens PE=2 SV=1 (B4DWU6_HUMAN)	0.474248	3.15*E*-05
37	Q14315	Filamin-C OS=Homo sapiens GN=FLNC PE=1 SV=3 (FLNC_HUMAN)	0.478864	3.98*E*-05
38	O75795	UDP-glucuronosyltransferase 2B17 OS=Homo sapiens GN=UGT2B17 PE=1 SV=1 (UDB17_HUMAN)	0.486547	5.80*E*-05
39	B2RTX2	Palladin, cytoskeletal associated protein OS=Homo sapiens GN=PALLD PE=2 SV=1 (B2RTX2_HUMAN)	0.491737	7.42*E*-05
40	G3 V144	SH3 and PX domain-containing protein 2B OS=Homo sapiens GN=SH3PXD2B PE=1 SV=1 (G3 V144_HUMAN)	0.492826	7.81*E*-05
41	Q05682	Caldesmon OS=Homo sapiens GN=CALD1 PE=1 SV=3 (CALD1_HUMAN)	0.498478	0.000101
42	Q99795	Cell surface A33 antigen OS=Homo sapiens GN=GPA33 PE=1 SV=1 (GPA33_HUMAN)	0.499929	0.000108
43	P21333	Filamin-A OS=Homo sapiens GN=FLNA PE=1 SV=4 (FLNA_HUMAN)	0.502317	0.000121
44	B7Z964	Sarcolemmal membrane-associated protein OS=Homo sapiens GN=SLMAP PE=1 SV=1 (B7Z964_HUMAN)	0.503373	0.000127
45	A0A142CHG9	GO2-q chimeric G-protein OS=Homo sapiens PE=2 SV=1 (A0A142CHG9_HUMAN)	0.503463	0.000127
46	A9LSU1	Type IV collagen alpha 1 (fragment) OS=Homo sapiens PE=2 SV=1 (A9LSU1_HUMAN)	0.504429	0.000133
47	B3KM36	cDNA FLJ10153 fis, clone HEMBA1003417, highly similar to BAG family molecular chaperone regulator 2 OS=Homo sapiens PE=2 SV=1 (B3KM36_HUMAN)	0.509395	0.000165
48	Q9UMK6	Dystrophin (fragment) OS=Homo sapiens GN=DMD PE=4 SV=1 (Q9UMK6_HUMAN)	0.517995	0.000238
49	P10645	Chromogranin-A OS=Homo sapiens GN=CHGA PE=1 SV=7 (CMGA_HUMAN)	0.528393	0.000364
50	A8K2W3	cDNA FLJ78516 OS=Homo sapiens PE=2 SV=1 (A8K2W3_HUMAN)	0.530959	0.000403

**Table 3:** Differential protein enrichment in 41 KEGG metabolic pathways.

n	Map ID	Map name	Number of protein	*P* value
1	map04672	Intestinal immune network for IgA production	5	7.81*E*-05
2	map05321	Inflammatory bowel disease (IBD)	5	0.000244
3	map05140	Leishmaniasis	7	0.001049
4	map04670	Leukocyte transendothelial migration	11	0.001234
5	map04510	Focal adhesion	14	0.001851
6	map05310	Asthma	4	0.003388
7	map05164	Influenza A	10	0.004899
8	map05144	Malaria	5	0.005231
9	map04514	Cell adhesion molecules (CAMs)	8	0.007151
10	map05145	Toxoplasmosis	8	0.007151
11	map00982	Drug metabolism-cytochrome P450	6	0.007464
12	map04973	Carbohydrate digestion and absorption	4	0.009718
13	map04972	Pancreatic secretion	7	0.010504
14	map05143	African trypanosomiasis	5	0.010539
15	map04530	Tight junction	9	0.013667
16	map04975	Fat digestion and absorption	4	0.014458
17	map04960	Aldosterone-regulated sodium reabsorption	4	0.014458
18	map05204	Chemical carcinogenesis	6	0.015534
19	map04974	Protein digestion and absorption	6	0.01907
20	map05150	Staphylococcus aureus infection	6	0.01907
21	map00983	Drug metabolism-other enzymes	4	0.020278
22	map05332	Graft-versus-host disease	4	0.020278
23	map04978	Mineral absorption	4	0.020278
24	map04933	AGE-RAGE signaling pathway in diabetic complications	6	0.023035
25	map05416	Viral myocarditis	6	0.023035
26	map05202	Transcriptional misregulation in cancer	5	0.023177
27	map05323	Rheumatoid arthritis	5	0.023177
28	map04261	Adrenergic signaling in cardiomyocytes	7	0.02526
29	map05330	Allograft rejection	4	0.027148
30	map05320	Autoimmune thyroid disease	4	0.027148
31	map05146	Amoebiasis	8	0.029455
32	map04612	Antigen processing and presentation	7	0.033293
33	map04940	Type I diabetes mellitus	4	0.034998
34	map04666	Fc gamma R-mediated phagocytosis	6	0.03738
35	map05412	Arrhythmogenic right ventricular cardiomyopathy (ARVC)	5	0.0413
36	map04971	Gastric acid secretion	5	0.0413
37	map04640	Hematopoietic cell lineage	4	0.043728
38	map04727	GABAergic synapse	4	0.043728
39	map04145	Phagosome	11	0.043902
40	map00980	Metabolism of xenobiotics by cytochrome P450	5	0.048381
41	map04970	Salivary secretion	5	0.048381

**Table 4:** The 14 differentially expressed proteins enriched in “focal adhesion” pathway.

n	Accession	GN	Description	*P* value
1	P12111	COL6A3	CO6A3_HUMANCollagen alpha-3 (VI) chain OS=Homo sapiens GN=COL6A3 PE=1 SV=5	0.0196709
2	Q15746	MYLK	MYLK_HUMANMyosin light chain smooth muscle OS=Homo sapiens GN=MYLK PE=1 SV=4	2.14273*E*-05
3	K7EM16	VASP	VASP_HUMANVasodilator-stimulated phospho OS=Homo sapiens GN=VASP PE=1 SV=3	0.000027793
4	Q14315	FLNC	FLNC_HUMANFilamin-C OS=Homo sapiens GN=FLNC PE=1 SV=3	0.000039772
5	P21333	FLNA	FLNA_HUMANFilamin-A OS=Homo sapiens GN=FLNA PE=1 SV=4	0.000120726
6	B7Z2N5	ACTN2	ACTN2_HUMANAlpha-actinin-2 OS=Homo sapiens GN=ACTN2 PE=1 SV=1	0.000524345
7	B7Z952	PARVA	PARVA_HUMANAlpha-parvin OS=Homo sapiens GN=PARVA PE=1 SV=1	0.00113605
8	P12814	ACTN1	ACTN1_HUMANAlpha-actinin-1 OS=Homo sapiens GN=ACTN1 PE=1 SV=2	0.00271591
9	A8K6A5	ITGA5	ITA5_HUMANIntegrin alpha-5 OS=Homo sapiens GN=ITGA5 PE=1 SV=2	0.0030417
10	Q03135	CAV1	CAV1_HUMANCaveolin-1 OS=Homo sapiens GN=CAV1 PE=1 SV=4	0.0047552
11	A0A024QZN4	VCL	VINC_HUMANVinculin OS=Homo sapiens GN=VCL PE=1 SV=4	0.0063742
12	A0A169TED2	PRKCA	KPCA_HUMAN kinase C alpha type OS=Homo sapiens GN=PRKCA PE=1 SV=4	0.0136269
13	P08572	COL4A2	CO4A2_HUMANCollagen alpha-2 (IV) chain OS=Homo sapiens GN=COL4A2 PE=1 SV=4	0.0140035
14	B4DTY8	ITGA1	ITA1_HUMANIntegrin alpha-1 OS=Homo sapiens GN=ITGA1 PE=1 SV=2	0.029126

## Data Availability

No data were used to support this study.
